# Detection performance and prognostic value of initial bone marrow involvement in diffuse large B-cell lymphoma: a single centre ^18^F-FDG PET/CT and bone marrow biopsy evaluation study

**DOI:** 10.2478/raon-2024-0004

**Published:** 2024-02-21

**Authors:** Andrej Doma, Katarina Zevnik, Andrej Studen, Veronika Kloboves Prevodnik, Gorana Gasljevic, Barbara Jezersek Novakovic

**Affiliations:** Department of Nuclear Medicine, Institute of Oncology Ljubljana, Ljubljana, Slovenia; Faculty of Medicine, University of Ljubljana, Ljubljana, Slovenia; Experimental Particle Physics Department, Jožef Stefan Institute, Ljubljana, Slovenia; Faculty of Mathematics and Physics, University of Ljubljana, Ljubljana, Slovenia; Department of Cytopathology, Institute of Oncology Ljubljana, Ljubljana, Slovenia; Faculty of Medicine, University of Maribor, Maribor, Slovenia; Department of Pathology, Institute of Oncology Ljubljana, Ljubljana, Slovenia; Division of Medical Oncology, Institute of Oncology Ljubljana, Ljubljana, Slovenia

**Keywords:** ^18^F-FDG PET/CT, Diffuse Large B-Cell Lymphoma, bone marrow, biopsy, overall survival

## Abstract

**Background:**

Detection of bone marrow involvement (BMI) in diffuse large B-cell lymphoma (DLBCL) typically relies on invasive bone marrow biopsy (BMB) that faces procedure limitations, while ^18^F-FDG PET/CT imaging offers a noninvasive alternative. The present study assesses the performance of ^18^F-FDG PET/CT in DLBCL BMI detection, its agreement with BMB, and the impact of BMI on survival outcomes.

**Patients and methods:**

This retrospective study analyzes baseline ^18^F-FDG PET/CT and BMB findings in145 stage II–IV DLBCL patients, evaluating both performance of the two diagnostic procedures and the impact of BMI on survival.

**Results:**

DLBCL BMI was detected in 38 patients (26.2%) using PET/CT and in 18 patients (12.4%) using BMB. Concordant results were seen in 79.3% of patients, with 20.7% showing discordant results. Combining PET/CT and BMB data, we identified 29.7% of patients with BMI. The sensitivity, specificity, positive predictive value (PPV), negative predictive value (NPV), and accuracy of PET/CT for detecting DLBCL BMI were 88.4%, 100%, 100%, 95.3%, and 96.5%, respectively, while BMB showed lower sensitivity (41.9%) and NPV (46.8%). The median overall survival (OS) was not reached in any gender subgroup, with 5-year OS rates of 82% (total), 84% (female), and 80% (male) (p = 0.461), while different International Prognostic Index (IPI) groups exhibited varied 5-year OS rates: 94% for low risk (LR), 91% for low-intermediate risk (LIR), 84% for high-intermediate risk (HIR), and 65% for high risk (HR) (p = 0.0027). Bone marrow involvement did not impact OS significantly (p = 0.979).

**Conclusions:**

^18^F-FDG PET/CT demonstrated superior diagnostic accuracy compared to BMB. While other studies reported poorer overall and BMI 5-year OS in DLBCL, our findings demonstrated favourable survival data.

## Introduction

Diffuse Large B-Cell Lymphoma (DLBCL) is the most common type of Non-Hodgkin lymphoma (NHL), comprising approximately 30–40% of all cases of NHL. DLBCL most frequently occurs in lymph nodes, but it can also affect other organs such as the spleen, liver, gastrointestinal tract, central nervous system, or bone marrow (BM).^1^ BM involvement (BMI) in DLBCL indicates a more advanced stage of the disease, playing a critical role in determining treatment strategies^2^ and predicting patient outcomes.^3^ The detection of BMI in DLBCL can be achieved through Positron Emission Tomography combined with Computed Tomography using the radiotracer fluorodeoxyglucose (^18^F-FDG PET/CT) and with BM aspiration and trephine biopsy (BMB). BMB has traditionally been considered as a gold standard for detecting BMI in DLBCL.^4,5^ BMB provides high diagnostic accuracy and allows for detailed morphological assessment of lymphoma cells, including their infiltration patterns within the bone marrow. Advantages of BMB include its ability to detect even focal or low-grade involvement, as well as its potential for identifying other concurrent bone marrow disorders or non-lymphoid malignancies.^6^ However, BMB is an invasive procedure associated with discomfort and possible complications.^7^ It may also be limited by the possible sampling errors, as the distribution of lymphoma cells can be heterogeneously within the BM, resulting in false negative BMB reports.^4^

^18^F-FDG PET/CT is a non-invasive imaging technique that provides functional and anatomical information, relying on the increased glucose metabolism of cancer cells including lymphoma cells.^8^ Several studies have evaluated the utility of ^18^F-FDG PET/CT in detecting BMI in DLBCL. ^18^F-FDG PET/CT can identify areas of increased glucose uptake, indicating the presence of lymphoma cells in the BM and ^18^F-FDG PET/CT has shown promising results in terms of sensitivity and specificity, with a high accuracy rate for detecting BMI in DLBCL patients.^9,10,11^

The aim of our study was to assess the performance of ^18^F-FDG PET/CT and its concordance with BMB results in patients with DLBCL and to evaluate consequential survival outcome.

## Patients and methods

### Patients population

Medical records of all patients, who were appointed to our institution with referral diagnosis of suspected DLBCL between January 2016 and December 2020 were retrospectively reviewed. Inclusion criteria were: histological confirmation of DLBCL; stage II to IV disease; BMB and ^18^F-FDG PET/CT prior to start of treatment. Exclusion criteria were: age <18 and >80 years; disease stage I; CNS involvement; history of prior or present other malignancies, including a low-grade lymphoma.

Patients were treated with 6 or 8 cycles of standard therapy according to local guidelines. The effect of treatment was assessed according to the Lugano classification with either CT or ^18^F-FDG PET/CT at the end of treatment, followed by the radiotherapy of the residual disease (determined by PET/CT), if applicable. All patients provided written informed consent for PET/CT and BMB and for the usage of medical data for the research purposes. This study has been approved by the Ethics Committee of the Institute of Oncology Ljubljana, number: ERIDEK-0104/2019and the National Medical Ethics Committee of the Republic of Slovenia, number: 0120-104/2021/3.

The collected clinical information included: sex, age, history of previous diseases, BMB report, stage, IPI score, serum LDH level, WHO performance status, type of therapy, date of progression, date of death.

### PET/CT

PET/CT acquisitions were performed using a Siemens Biograph mCT40 PET/CT, according to current guidelines.^12^ In short, all patients fasted for the last 6 hours before the examination. The blood sugar level prior to the injection of ^18^F-FDG was <7 mmol/L and if necessary, i.v. Insulin was applied. FDG activity of 3.7 MBq/kg was administered intravenously1 hour prior to imaging. A tip-of-the-head to mid-thigh PET/CT acquisition was performed with the following settings: the system regulated current and voltage for each subject based on the reference kV value of 100 kV and reference mAs value of 80 mAs; beam width: 16 × 1.2 mm; Pitch: 1.2; PET acquisition time 2 min/bed position.

All ^18^F-FDG PET/CT images were assessed by two experienced nuclear medicine physicians, blinded for BMB reading, and conclusions for discrepant cases were made with consensus.

### Bone marrow biopsy

According to the International Guidelines, BM trephine biopsy and aspiration were performed either by medical oncologist or by surgeon (performing the lymph node biopsy) according to standard protocol in all patients included in the study.^13^ All BM trephine biopsies were fixed in 10% buffered formalin overnight. Each specimen was then cut into two halves parallel to the longitudinal axis. One half of the specimen was embedded into a resin and stained by Giemsa, H&E, chloroacetate esterase, PAS, Pearls and Gomory. The other half was decalcified in EDTA and used for immunohistochemical analysis. Standard immunohistochemistry panel consisting of CD20 (DAKO, 1:500), CD3 (DAKO, 1:400), PAX-5 (DAKO, 1:40), MIB-1 (DAKO, 1:200), MPO (DAKO, 1:4000), CD61 (Cell Marque, 1:200), CD71 (Roche, ready-to-use) was applied and was executed on 2–4 μm thick FFPE tissue sections dried at 56°C for 2 h using the fully automated IHC staining platform Benchmark Ultra (Manufacturer Ventana ROCHE Inc., Tucson, AZ, USA). Pathohistological examination of BM was performed by a skilled hematopathologist. From all BM aspirates, May-Grunwald-Giemsa smears were prepared for microscopic evaluation and flow-cytometric immunophenotyping was carried out. Before flow-cytometric analysis, the cell count was automatically determined using the Sysmex XP-300 hematological analyser (Sysmex). Sample preparation for flow-cytometric analysis was carried out as previously described by our group.^14,15^ Antibodies presented in Table 1 were divided in 9 tubes and half a million cells were put in each tube. After 20 min incubation, erythrocyte lysis was carried out using a commercial lysing solution (BD Biosciences). Flow-cytometric data were acquired by a 10-color BD FACSCanto™ II Flow Cytometer and FACSDiva 8.0.2 software (BD Bioscience).

**TABLE 1.** The six-colour antibody panel used for flow-cytometric immunophenotyping of bone marrow (BM) aspirates

**Tube**		**FITC**	**PE**	**PerCP-Cy5,5**	**APC**	**PE-Cy7**	**APC-Cy7**
1.	mAb	κ	λ	CD19	CD5	CD10	CD45
	V^é^	5 μl	5 μl	5 μl	3 μl	2 μl	3 μl
2.	mAb	CD34	CD117	CD33	HLA-DR	CD14	CD45
	V^é^	3 μl	3 μl	2 μl	2 μl	3 μl	3 μl
3.	mAb	CD3	CD56	CD5	CD20	CD19	CD45
	V^é^	1 μl	3 μl	3 μl	6 μl	3 μl	3 μl
4.	mAb			CD19			CD45
	V^é^			5 μl			3 μl
5.	mAb	FMC7	CD23	CD19	CD5	CD10	CD45
	V^é^	6 μl	6 μl	5 μl	3μl		
6.	mAb	CD52	CD11c	CD19	CD38		CD45
	V^é^	3 μl	5 μl	5 μl	3 μl		3 μl
7.	mAb	CD103	CD22	CD19	CD25		CD45
	V^é^	5 μl	4 μl	5 μl	2 μl		3 μl
8.	mAb	CD38	CD56	CD19	CD138		CD45
	V^é^	5 μl	3 μl	5 μl	5 μl		3 μl
9.	mAb	CD4	CD8	CD3	CD7	CD5	CD45
	V^é^	3 μl	4 μl	3 μl	2 μl	5 μl	3 μl

APC = allophycocyanin; APC-Cy7 = allophycocyanin-cyanine 7; FITC = fluorescein isothiocyanate; mAb = monoclonal antibody; PE = phycoerythrin; PerCP-Cy5.5 = peridinin-chlorophyll-protein-complex-Cy5.5; PerCP = peridinin-chlorophyll-protein-complex; PE-Cy7 = phycoerythrin-cyanine7; V = volume; V^é^ = the volume of the antibodies was adjusted according to the results of titration measurements

### PET scan interpretation

BMI on ^18^F-FDG PET/CT was evaluated using semi-quantitative analysis by measuring the maximum standardized uptake values (SUVmax) in BM DLBCL infiltrates and normal non-infiltrated liver and comparing the two values. Additionally, a pattern of BM uptake of ^18^F-FDG was considered in the analysis.

Positive BMI on ^18^F-FDG PET/CT was defined in case of focal or multifocal pattern of increased BM ^18^F-FDG uptake, greater than normal liver uptake, which could not be explained as a benign aetiology by CT or clinical correlation. Negative BMI on ^18^F-FDG PET/CT was considered in case of diffuse BM ^18^F-FDG uptake, irrespective of the degree of uptake, in case of no detectable uptake in BM, and in case of a putative benign aetiology of increased ^18^F-FDG uptake.^16,17^

### BMI determination

BMB was considered the reference standard to detect BMI. Concordant positive and negative ^18^F-FDG PET/CT BM findings and BMB results were considered as true positive and true negative results, and negative ^18^F-FDG PET/CT scan results in patients with a positive DLBCL BMB were considered as false-negative results.

Discordant ^18^F-FDG PET/CT BM findings and BMB results in which PET/CT results were positive and BMB results were negative were correlated with evaluation PET/CT at the end of the treatment: BM was defined as positive on ^18^F-FDG PET CT in case of disappearance or decrease of activity comparable to nodal DLBCL infiltrates on evaluation PET/CT scans, and negative in case of unchanged persistent activity on post-treatment evaluation PET/CT scans, when the activity of nodal DLBCL infiltrates would noticeably decrease.

### Statistical analysis

Statistical analysis of sensitivity, specificity, accuracy, positive and negative predictive values of BMB and ^18^F-FDG PET/CT for detection of BMI was performed. Survival analysis was performed with Kaplan-Meier curves and log-rank tests, differences between groups for the continuous variables were compared with Mann-Whitney U test, and the Chi-squared test was used to measure the association or independence between categorical variables; p values <0.05 were considered significant. Statistical analysis was performed using MedCalc 19.2.6 (MedCalc Software, Belgium).

## Results

Medical records of 507 patients were retrospectively reviewed. DLBCL was histologically confirmed in 371 patients. Of those, 194 patients were excluded from analysis (stage I disease n = 33; CNS involvement n = 15; palliative care n = 11; no FDG PET/CT performed n = 126; age >80 years n = 9). Another 32 patients did not have a BMB. Thus, 145 patients were included in the analysis. Patients’ characteristics are presented in Table 2.

**TABLE 2.** Patients’ characteristics

**Age (median, range), years**	65 (20–79)	
**Gender, female/male,** n (%)	64 (44.1%)/81 (55.9%)	
	**BMI −**	**BMI +**
**Age (median) [years]; BMI−: BMI+**	66 (25–79)	63(20–78); p = 0.524
**Gender, female/male,** n;	48/54	16/27; p = 0.277
**IPI score,** n (%)		
**IPI Low risk group (LR):** 33 (22.8%)	32	1
**IPI Low-intermediate risk group (LIR):** 32 (22.1%)	22	10
**IPI High-intermediate risk group (HIR):** 33 (22.8%)	24	9
**IPI High risk group (HR):** 47 (32.4%)	24	23
**Stage at diagnosis,** n (%)	II: 39 (26.9%)	
	III: 19 (13.1%)	
	IV: 87 (60.0%)	
**Chemotherapy regimen,** n (%)	R-CHOP: 119 (82.1%)	
	R-EPOCH: 9 (6.2%)	
	R-ACVBP: 6 (4.1%)	
	RCOEP: 3 (2.1%)	
	Other: 8 (5.5%)	
**Radiotherapy after chemotherapy,** n (%)	52 (35.9%)	
**Death in 60 months follow-up period,** n (%)	29 (20.0%)	
**Bone marrow involvements present-overall,** n (%)	43 (29.7%)	
**Extranodal sites:** 0, n (%)	26 (17.9%)	
**Extranodal sites:** 1, n (%)	48 (33.1%); of those BMI n = 11 (7.6%)	
**Extranodal sites more than 1,** no. (%)	71 (49.0%)	

BMI = bone marrow involvement; IPI = international prognostic index; R-ACVBP = rituximab, doxorubicin, cyclophosphamide, vindesine, bleomycin, prednisone (R-ACVBP); R-CEOP = rituximab, cyclophosphamide, doxorubicin, vincristine, and prednisone; R-CHOP = rituximab, cyclophosphamide, doxorubicin, vincristine, prednisolone (a steroid) – (pred-ni-suh-lown); R-EPOCH = rituximab, etoposide, prednisone, vincristine, cyclophosphamide, hydroxydaunorubicin

DLBCL BMI was detected in 38 patients (26.2%) by PET/CT and in 18 patients (12.4%) by BMB; concordant results between PET/CT and BMB were observed in 115 (79.3%) patients; in 102 (70.3%) negative and 13 (9.0%) positive. Discordant results were seen in 30 (20.7%) patients; in 25 (17.2%) with true positive PET/CT and false negative BMB and in 5 (3.4%) with false negative PET/CT and true positive BMB.

Through a combined analysis of PET/CT and BMB data, we determined the absence of BMI in 102 patients (70.3%) and its presence in 43 patients (29.7%), with all 30 discordant results being acknowledged as BMI positive.

The sensitivity, specificity, positive predictive value (PPV), negative predictive value (NPV), and accuracy for ^18^F-FDG PET/CT for the detection of DLBCL BMI was 88.4% (95% confidence interval [Cl]; 74.9–96.1), 100% (95% Cl; 96.4–100), 100% (95% Cl; 0–0), 95.3% (95% CL; 89.9–97.9) and 96.5% (95% Cl; 92.1–98.9). Regarding the BMB, the sensitivity, specificity, PPV, NPV, and accuracy for the detection of DLBCL BMI was 41.9% (95% Cl; 27.0–57.9), 100% (95% Cl; 84.6–100), 100% (95% Cl; 0–0), 46.8% (9 5% Cl; 40.6–53.1), and 61.5% (95% Cl; 48.6–73.3).

No significant association was found between the gender and BMI (χ^2^[1] = 1.182, p = 0.277), however statistically significant association was observed between different IPI groups and BMI (χ^2^[3] = 19.718, p = 0.0002).

**FIGURE 1.**
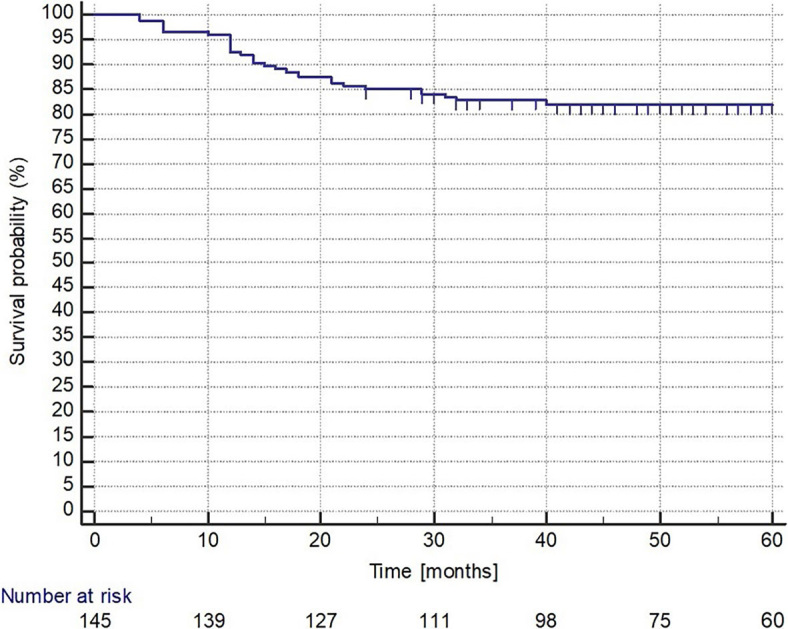
Overall survival.

**FIGURE 2.**
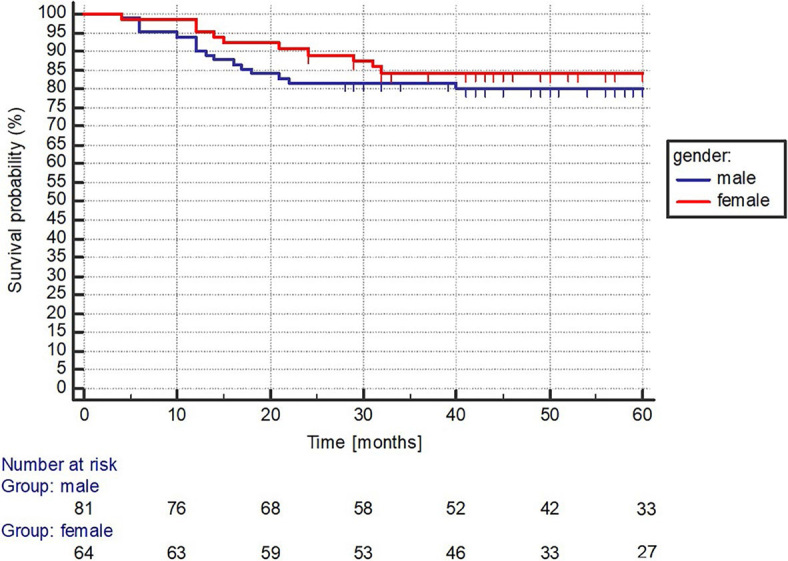
Gender based survival analysis.

**FIGURE 3.**
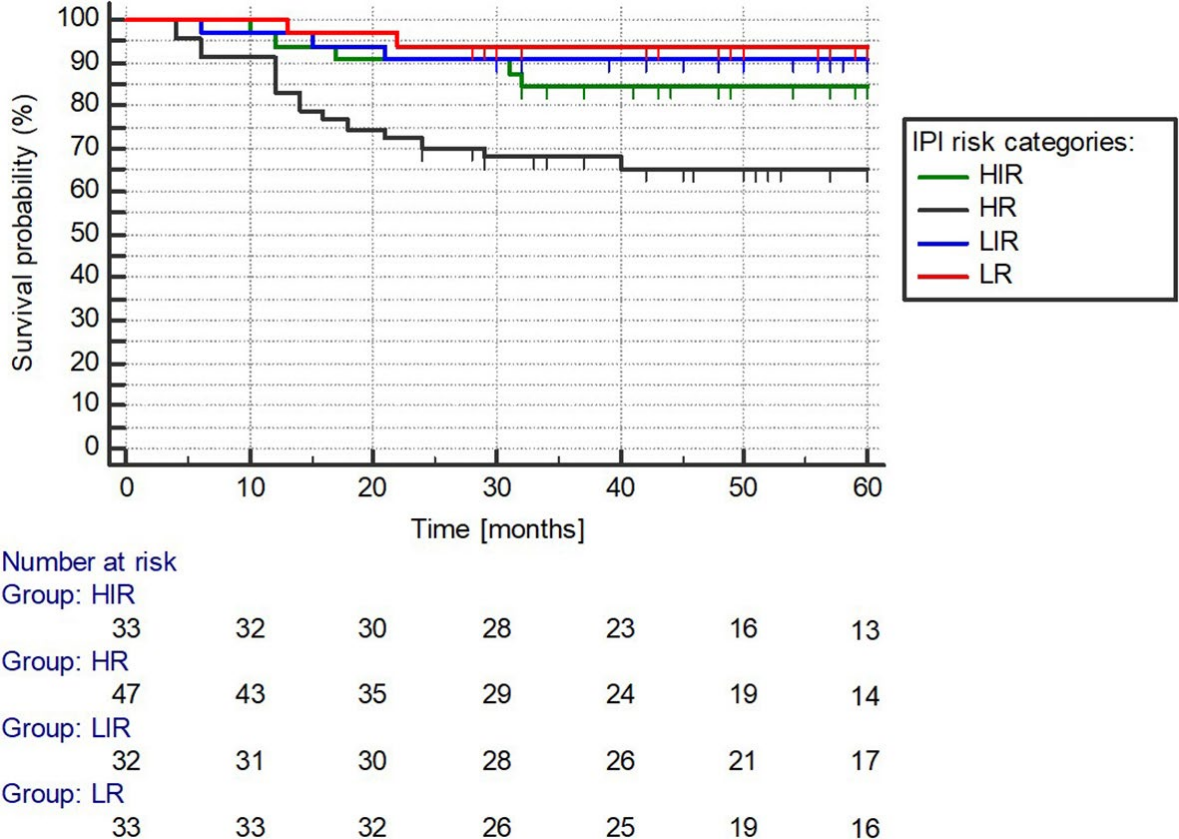
International prognostic index (IPI) survival analysis. HR = high risk; HIR = high-intermediate risk; LIR = low-intermediate risk; LR = low risk

The median OS has not been reached in any of the total (Figure 1), female and male group with a 5-year OS rate of 82%, 84%, and 80%, respectively (p = 0.461) (Figure 2). The median OS has not been reached in any of the IPI LR, LIR, HIR, and HR groups with a 5-year OS rate of 94%, 91%, 84%, and 65%, respectively, while the association between the IPI groups and the 5-year OS rates was statistically significant (P = 0.0027) (Figure 3).

**FIGURE 4.**
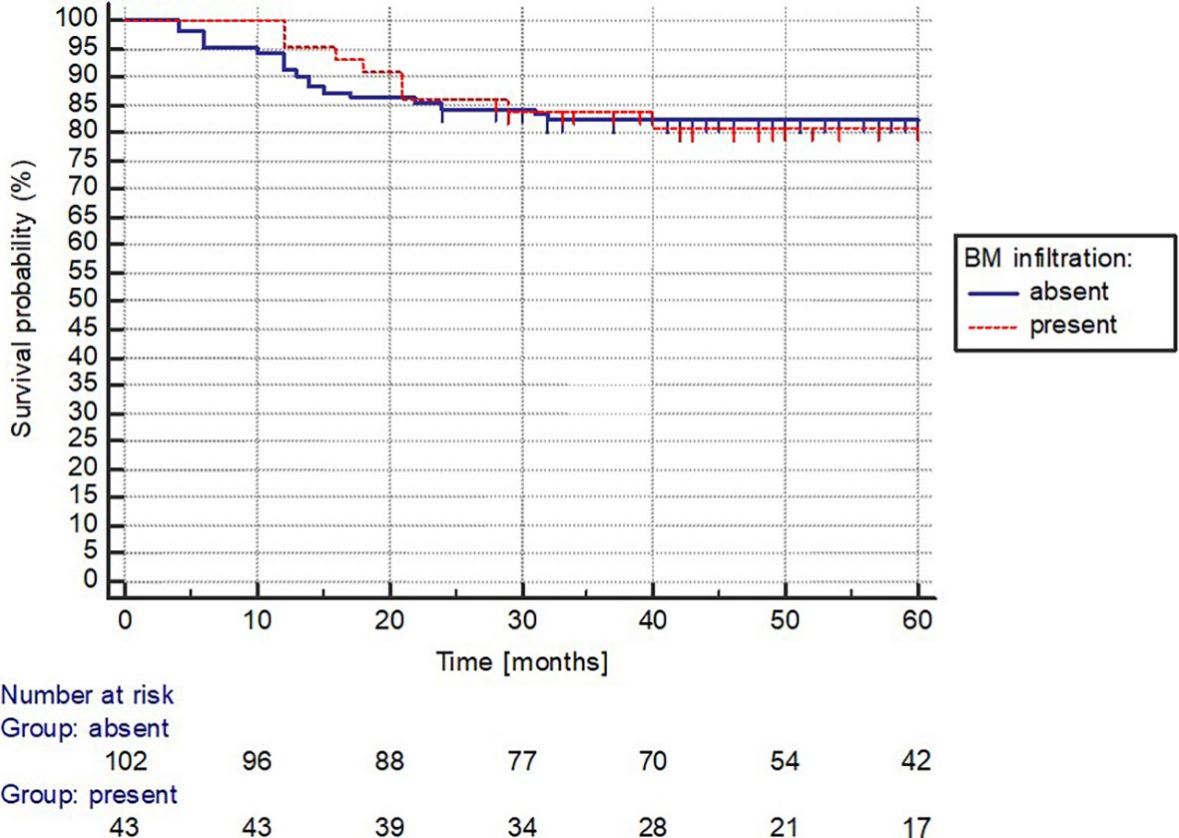
Bone marrow involvement status survival analysis. BM = bone marrow

The median OS was not yet reached in the BM non-involved (absent) and BM involved (present) groups with a 5-year OS rate of 82%, and 81%, respectively (p = 0.979) (Figure 4).

## Discussion

The presence of BMI in DLBCL upstages the disease to stage IV, underscoring the critical importance of accurately assessing BMI for determining appropriate treatment strategies and predicting patient outcomes.^18^

Historically, BMB has been regarded as the gold standard for assessing BMI. However, recent studies have demonstrated superior results with ^18^F-FDG PET/CT. Blind, unifocal BMB procedures come with several drawbacks, including the potential to miss a patchy pattern of BMI.^19^ Additionally, BMB can induce discomfort, pain, and potential bleeding in patients.^20^

In contrast, ^18^F-FDG PET/CT, as a whole-body imaging modality, is known for providing highly accurate staging data across various malignancies while maintaining safety and causing only moderate levels of anxiety for patients.^21^ As a result, current guidelines no longer recommend BMB when a PET/CT scan reveals bone or marrow involvement, indicating advanced-stage disease. However, BMB is still advisable in cases where PET results are negative, when a shift in BMI status would impact prognosis and treatment decisions, and to exclude discordant low-grade lymphoma.^18^

In our retrospective study, we found that ^18^F-FDG PET/CT exhibited significantly higher accuracy compared to BMB as a diagnostic modality. This observation aligns with several other studies^5,9,22,23,24^, that reported superior sensitivity of ^18^F-FDG PET/CT over BMB, with only one study^25^ indicating similar accuracy between the two diagnostic methods.

Our study’s findings regarding sensitivity and specificity are consistent with the results of a systematic review conducted by Almaimani *et al.* in 2022, which analysed 20 studies involving 2336 DLBCL patients.^26^ The authors reported combined sensitivity and specificity values of 77% and 92% for ^18^F-FDG PET/CT and 47% and 100% for BMB, respectively.

In our dataset, ^18^F-FDG PET/CT produced false-negative results in 5 out of 43 (11.6%) BMI+ patients which is consistent with the findings of a previous large multicentre study by Pelosi *et al.*^24^ Our data support the notion that ^18^F-FDG PET/CT can effectively replace BMB for determining BMI status in DLBCL patients.

False positive BMI findings on FDG PET/CT can be caused by a variety of factors, such as focal uptake of other types of cancer or septic diseases, and diffuse BM uptake due to inflammation.^27,28^ By implementing rigorous inclusion and interpretation criteria, we achieved perfect specificity and a positive predictive rate of 100%.

Several studies have reported poor OS rates in DLBCL patient cohorts, with BMI being associated with further reductions in OS. Recent studies by Yao *et al.* and Alonso-Alvarez *et al.* demonstrated statistically significant differences in 5-year OS rates between BMI- and concordant BMI+ patients (67.7% *vs*. 42% and 72% *vs*. 51%, respectively).^29,30^ However, our study showed significantly better OS outcomes, with 5-year OS rates of 94%, 91%, 84%, and 65% for IPI LR, LIR, HIR, and HR groups, respectively. Interestingly, we did not find a statistically significant difference in 5-year OS between BMI- and BMI+ groups.

The discrepancies in OS results among studies could be attributed to variations in patient characteristics. For example, our study excluded patients with CNS involvement, whereas Alonso-Alvarez’s study included such cases.^30^ Other differences include variations in the frequency of IPI high-risk patients and the inclusion of stage I disease. Additionally, discrepancies may arise from the different methods used to determine BMI status; some studies relied solely on BMB results, while our analysis incorporated both BMB and PET/CT-based BMI assessments.

Our study’s impressive survival data is consistent with previous reports from a large cohort of patients treated at our institution between 2004 and 2013, where 5-year OS ranged from 86% in IPI LR patients to 45% in IPI HR patients.^31^ The high-quality clinical care provided by our dedicated lymphoma department likely contributes to these favourable outcomes.

It is important to acknowledge the limitations of our study, inherent from its retrospective design and potential selection bias. Future research on the prognostic value of baseline PET/CT in lymphoma, including the impact of BMI on survival, should move beyond binary qualitative PET/CT results (present/absent) and incorporate tumour burden analysis using advanced radiomics quantitative segmentation methods such as metabolic tumour volume (MTV) and total lesion glycolysis (TLG).

## Conclusions

In conclusion, the findings of our study suggest that ^18^F-FDG PET/CT may be a more accurate method for detecting BMI in DLBCL patients, and that BMI may not be a significant prognostic factor for OS in this population.
